# Argyrophilic grain disease: An underestimated tauopathy

**DOI:** 10.1590/S1980-57642015DN91000002

**Published:** 2015

**Authors:** Roberta Diehl Rodriguez, Lea Tenenholz Grinberg

**Affiliations:** 1 MD, Department of Pathology, University of São Paulo, SP, Brazil; Brazilian Aging Brain Study Group, LIM-22, University of São Paulo, São Paulo, Brazil; 2 MD, PhD, Department of Pathology, University of São Paulo, SP, Brazil; Memory and Aging Center, Department of Neurology and Pathology, University of California, San Francisco; Brazilian Aging Brain Study Group, LIM-22, University of São Paulo, São Paulo, Brazil.

**Keywords:** argyrophilic grain disease, dementia, tauopathies, neurodegenerative diseases, pathology, postmortem

## Abstract

Argyrophilic grain disease (AGD) is an under-recognized, distinct, highly frequent sporadic tauopathy, with a prevalence reaching 31.3% in centenarians. The most common AGD manifestation is slowly progressive amnestic mild cognitive impairment, accompanied by a high prevalence of neuropsychiatric symptoms. AGD diagnosis can only be achieved *postmortem* based on the finding of its three main pathologic features: argyrophilic grains, oligodendrocytic coiled bodies and neuronal pretangles. AGD is frequently seen together with Alzheimer's disease-type pathology or in association with other neurodegenerative diseases. Recent studies suggest that AGD may be a defense mechanism against the spread of other neuropathological entities, particularly Alzheimer's disease. This review aims to provide an in-depth overview of the current understanding on AGD.

## Abstract (pt)

A doença com grãos argirofílicos (DGA) é uma taupatia esporádica distinta, bastante frequente, porém pouco reconhecida com uma prevalência atingindo 31,3% em centenários. A manifestação clínica mais comum da DGA é de um comprometimento cognitivo de lenta evolução associado a uma alta prevalência de sintomas psiquiátricos. O diagnóstico de DGA é possível somente com a análise do encéfalo *post-mortem* com os achados das três principais alterações patológicas: grãos argirofílicos, corpúsculos em embrião e pré-emaranhados neuronais. A DGA é, frequentemente, associada com alterações patológicas do tipo Alzheimer. Estudos recentes sugerem a AGD como um mecanismo de defesa contra a propagação de outras alterações neuropatológicas, em especial a doença de Alzheimer. Essa revisão almeja proporcionar uma visão geral do conhecimento atual sobre a DGA.

## INTRODUCTION

Argyrophilic grain disease (AGD) is highly frequent, but still under-recognized neurodegenerative condition.^1^ AGD has proven the second-most-common neurodegenerative disease after Alzheimer's disease (AD) in several studies.^2-6^ The incidence of AGD increases significantly with age and its prevalence has been estimated to range from 9.3% in 65 year-olds to 31.3% in centenarians.^4,7-9^ Although AGD is more prevalent in older individuals, it is not exclusively seen in the elderly. Ishihara et al. reported a 54-year-old man with a neuropathological diagnosis of AGD presenting frontotemporal dementia at age 49 years of age. Saito et al. detected AGD in a 56-year-old man from a series of 1,405 autopsies.^6,10^ In the series of the Brain Bank of the Brazilian Aging Brain Study Group, 6.7% of individuals aged between 50 and 60 had AGD.^11^

AGD was first reported in 1987, by Braak and co-workers as a progressive, late-onset neurodegenerative disease characterized by small spindle- or comma-shaped, silver stain positive lesions in neuronal processes, referred to as argyrophilic grains (AG).^12^ Subsequently, studies revealed hyperphosphorylated-tau protein as a major AG component. AGD is frequently seen overlapping with AD pathology, making it even more difficult to investigate the role of AGD in cognition.^2,13-15^ Consequently, many have recognized AGD as a distinctive pathological entity only recently.^16-23^ The first descriptions characterized AGD as a novel neuropathological entity in patients with progressive late-onset dementia who lacked sufficient numbers of Alzheimer-type lesions to warrant the morphological diagnosis of Alzheimer disease,^12,24,25^ associated with dementia or otherwise.^4,5,26-33^

At any rate, AGD is virtually unknown to the clinical community because most cases do not present any known distinctive clinical symptoms.^4,5,9,26-29,31-34^

**Clinical presentation.** Clinicopathological studies failed to show a distinctive clinical or imaging phenotype associated with AGD, and currently, diagnosis is only possible through *postmortem* brain analysis.^17^

A very slowly progressive mild cognitive impairment (MCI) is the most common AGD manifestation.^4,13,36^ Saito et al. in a study investigating pathological changes underlying MCI, investigated 545 serial autopsy cases from a general geriatric hospital. They found neurodegenerative pathology in 57% of MCI cases (19% AD and 18% AGD). The authors then extended the study to also include dementia cases and found AD in 45% and AGD in 18% of the cases. Their findings suggested that a higher relative frequency of beta-amyloid-independent tauopathies, such as AGD, in the MCI group is probably related to the more benign course of such diseases.^36^

Some studies have associated AGD with neuropsychiatric symptoms. AGD subjects present more personality changes and emotional imbalance preceding memory failure than age-matched controls.^10,13,37-39^ There is evidences that at early and mild stages of the disease, emotional and personality changes are the dominant clinical features of AGD, reflecting the prominent involvement of the limbic system.^6,40,41^ Saito et al. reported that AGs are initially found in the temporo-amygdaloid junction, and that the distribution of AGs followed a stereotypic regional pattern of spread reflecting an antero-posterior gradient in putative AGD progression. A study comparing Parkinson's disease patients with and without AGD, and studies showing a high prevalence of AGD in individuals with late-onset schizophrenia and delusional disorders, corroborate the association of AGD with psychiatric symptoms.^42,43^ Asaoka et al. reported an AGD case exhibiting delusions and hallucinations at clinical presentation.^37^

A few reports implicate AGD as the underlying condition in patients featuring clinical frontotemporal dementia. In such cases, AGD pathology is widespread in the brain, as opposed to the majority of AGD cases where involvement is restricted to limbic structures. Tsuchiya et al. reported an 89-year-old female featuring temporal frontotemporal dementia (previously known as Pick's disease), amnesia, wandering, personality changes characterized by unusual rude, stubborn and egocentric behavior.^40^ Maurage et al. reported two cases fulfilling revised criteria for possible behavioral-variant frontotemporal dementia (bvFTD): a 76-year-old female with cognitive impairment, obsession, apathy, carbohydrate craving; and a 79-year-old female with cognitive slowing, apathy, memory impairment, logorrhea and carbohydrate craving.^44,45^ Finally, Ishihara et al. described a 54-year-old male presenting personality changes and abnormal stereotypical behavior, disinhibition and environment-dependent behavior characterizing bvFTD. All these cases presented overlapping mild Alzheimer-type pathology.^10^

AGD can also be present in patients without cognitive impairment. Knopman et al. showed AGD in 31% of a series of cognitively normal subjects.^46^ The anatomical substrate for cognitive impairment in AGD is unclear and studies have implicated the entorhinal cortex, hippocampus, temporal association cortex and amygdala.^47^ Tolnay et al. examined the limbic area in 19 demented patients with AGD and 16 non-demented patients with AGD. They demonstrated that the caudal part of hippocampal sector CA1 was found to be significantly more affected in demented AGD than in non-demented AGD. On the contrary, AG burden in rostral CA^1^ was similar in both demented and non-demented cases, suggesting that early subclinical lesions may begin in the anterior part of the hippocampal formation.^9^ Recently, the severe degeneration of the ambient gyrus was associated with the development of dementia in AGD cases.^41^

**Biochemical features.** Tau is the major neuronal microtubule-associated protein (MAP) encoded by the microtubule-associated protein gene (MAPT). The tau gene is located at chromosome 17q^21^. In human adults, tau features six different isoforms due to alternative mRNA splicing of the exons 2, 3 and 10. The isoforms differ by the presence or absence of 29- or 58- aminoacid inserts in the N- terminal portion of the molecule or by the inclusion or otherwise of the 31 aminoacid repeat in the C- terminal part that is encoded by exon 10 of the MAPT. The inclusion of exon 10 generates an isoform with four microtubule-binding domains (4R), while the absence of this inclusion produces an isoform with three microtubule binding domains (3R). In the adult healthy brain, there is an equal proportion of 3R and 4R forms.^48,49^ AGD features predominant deposition of 4R-tau, as do progressive supranuclear palsy (PSP) and corticobasal degeneration (CBD), whereas AD is characterized by the simultaneous presence of both 3R and 4R tau proteins.^44,50^

Tau protein promotes assembly of and stabilizes microtubules, one of the major structural components of the axonal transport and neurotransmission machinery. Abnormal phosphorylation of tau decreases its binding capacity to tubulin, leading to microtubule disorganization with protein self-polymerization and aggregation. Tau phosphorylation is regulated by tau kinases, phosphatases and changes in its conformational state.^48,51^ The mechanism by which tau protein becomes nonfunctional remains unknown, but abnormal posttranslational modifications other than phosphorylation may play an important role in this process.^35,52^ Recent studies have shown that increased tau acetylation could impair tau interactions with microtubules and contribute to tau aggregation. Evidence suggests that tau acetylation affects its degradation by polyubiquitination inhibition.^22,35,53^ Grinberg et al. demonstrated that tau acetylation is not present in AGD unlike in other common and rare tauopathies such as AD, Pick's Disease, PSP, chronic traumatic encephalopathy and familial tauopathies. The lack of tau acetylation in AGD and the fact that hyperphosphorylation alone does not necessarily promote tau aggregation and cellular dysfunction suggest that AGD may be a protective mechanism against spread of other tauopathies rather than a harmful entity.^22^

Llieva et al. showed that the expression of apoptosis inducing factor, a multifunctional mitochondrial protein involved in oxidative stress and mitochondrial structure, is increased in both AGD and AD, but altered expression of components of the respiratory chain markers modulating mitochondrial biogenesis was selectively affected in AGD.^16^

**Neuropathological features.** Macroscopically, AGD brains may be unchanged or only mildly atrophic, not differing from age-matched controls.^4,5^ Tolnay et al. only found mild temporal lobe atrophy in a few of their 41 AGD cases.^9^

Microscopically, AGD is characterized by three main pathologic features:

[1] AGs, the pathologic hallmark lesion;
[2] oligodendrocytic inclusions called coiled bodies, and
[3] neuronal intracytoplasmic tau-positive granular inclusions called pretangles.

Associated pathologic lesions include balloon neurons and bush-like astrocytes.^8,19,47,50,54^ Although coiled bodies and pretangles are not exclusive to AGD, they are positive for acetylated tau in all other tauopathies, yet not in AGD.^22^

**Figure 1:**
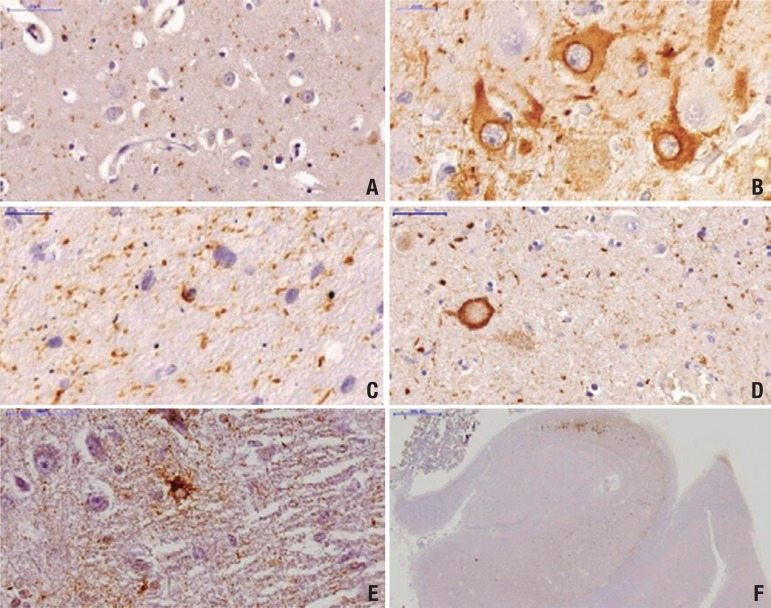
Tau pathological features in an AGD case immunostained with PHF-1 antibody. [A] Grains in the CA1 subfield of the hippocampus; [B] Pretangles with perinuclear halo in the CA2 subfield of the hippocampus; [C] Coiled-body in the CA1 subfield of the hippocampus; [D] Balloon neuron in amygdala (arrow); [E] Bush-like astrocyte in amygdala; [F] Prominent involvement of CA2 subfield of the hippocampus with sparing of the CA1 subfield. *Scale bars* represent 50 µm in A, D, E; 20 µm in B,C and 200 µm in F.

*Argyrophilic grains:* small spindle- or comma- shaped structures found within neuronal processes mainly in the dendrites and dendritic spines (Figure 1).^12,26,30,47^ AGs predominates in layers II and III of cerebral cortex.^12^ They exhibit a typical distribution pattern, more abundant within the limbic system, mainly in the CA^1^ sector of the hippocampus, subiculum, entorhinal and transentorhinal cortex, and adjacent temporal isocortex.^5,41^ The amygdala and hypothalamic lateral nucleus are the most severely affected subcortical structures.^5,25^ The size of AGs apparently increases with advancing stage, although areas with severe neuronal loss became devoid of AGs.^41^

AGs are not immunoreactive for antibody 12E8, against phosphorylated-tau at Ser 262/356, suggesting that different phosphorylation at this site is a late event occurring only in compact neurofibrillary lesions.^55^

*Coiled bodies:* oligodendrocytic inclusions are largely restricted to the white matter underlying the limbic cortex (Figure 1). These lesions are present in other 4R tauopathies such as PSP and CBD, but have a different anatomic distribution.^50^

*Pretangles:* these neuronal cytoplasmic tau-positive inclusions are Gallyas and ubiquitin-negative (Figure 1). Although they are distributed similarly to AGs,^8,18^ their presence is ubiquitous in the CA2 sector of the hippocampus, a region extremely resistant to AD-type changes.^9,30^

*Balloon neurons:* are characterized by swelling of the perikarya and loss of Nissl substance of pyramidal neurons. They are found in the brains of different neurodegenerative disorders, but not in normal brains (Figure 1). In AGD, balloon neurons are more frequent in the amygdala and do not correlate with the density of AGs or the severity of associated lesions.^34,54,56^

*Bush-like astrocytes:* seen in the amygdala and anterior entorhinal cortex of AGD cases (Figure 1). Despite being positive for hyperphosphorylated-tau, they do not display fibrils as evidenced by the absence of Gallyas positive staining.^19^ In addition, they are not a prominent feature of AGD.^47,50,57^ Botez et al. suggest that, together with other features, the presence of bush-like astrocytes may help to differentiate AGD from AD.^19^

Two different staging systems are available for staging AGD. Saito et al. suggest a 3-point system taking into consideration AGs, balloon neurons, bush-like astrocytes and coiled-bodies.^6^ The earliest changes are evident in the ambiens gyrus, followed by the anterior and posterior medial temporal lobe, and finally the septal area, anterior cingulate gyrus and insular cortex in later stages. Ferrer et al. propose a 4-point scale system considering AG distribution only. Involvement of anterior entorhinal cortex, basolateral nuclei of amygdala and hypothalamic lateral tuberal nucleus represent early stages while neocortex and brainstem become involved in the last stage.^47^

Thal et al. studied the pattern of alterations in temporo-entorhinal association neurons in seven brains (two with mild AD-related pathology without cognitive impairment, one severe AD-related pathology without cognitive impairment, one demented AD, and three AGD cases with dementia) using the *postmortem* Dil tracing technique. They showed a disconnection of association fibers between the entorhinal and the adjacent temporal neocortices in the AD case and in one non-demented case with severe AD- related pathology. By contrast, all the AGD cases with cognitive impairment lacked obvious disconnection between the entorhinal and the temporal neocortices. However, the AGD cases displayed dendritic tree abnormalities when compared to the non-demented AD-related pathology and to the non-demented case with severe AD- related pathology. These findings suggest differences in the pattern of neurodegeneration between AGD and AD. Dementia in AD may be related to major anatomical disconnection, whereas dementia in AGD may be related to dendritic degeneration with preserved axonal connections.^23,58^

*Other pathological changes:* One study suggests prominent superficial laminar spongiosis in cortical areas rich in AGs, a finding not confirmed in our observations.^8^

Because of the high overlap between AD-type pathology and AGD, some advocate that the latter is part of the AD spectrum. However, fundamental differences can be noted between these two entities. [1] the AG spread pattern does not fit under the neurofibrillary staging system.^59^ Davis et al. found an age-associated increase of AGD in a study involving 20 AGD cases and controls. They found no association between AGs and AD-type neurofibrillary tangles or neuropil threads in the CA1 sector of the hippocampus. Finally, the number of neurofibrillary tangles and neuropil threads in AGD did not differ across the cognitive decline spectrum;^60^ [2] Unlike in AD, the hippocampal sector CA2 and dentate gyrus are very vulnerable to AGD (Figure 1). Likewise, the hypothalamus shows a different vulnerability pattern to AGD with early and prominent involvement of lateral tuberal nuclei and relative resistance of tuberomammillary nuclei. In AD, the tuberomammillary nuclei are severely affected by neurofibrillary tangles.^61,62^ The profile of Aβ deposition in AGD resembles that of cognitively normal elderly rather than of AD.^31^

**Associated neurodegenerative diseases.** AGs may resemble neuropil threads cut transversally, hampering diagnosis of AGD in patients harboring moderate-to-severe AD pathology.^24,28^ Braak and Braak, in a series of 2661 unselected autopsies of subjects aged 27 to 96 years, observed 125 cases of AGD as compared to 146 cases of AD stage V to VI. Most of the cases with AGD and severe progressive dementia showed only mild concomitant AD pathology corresponding to Braak stages I to III and only 12.5 % showed Braak stage V.^4^ The lower prevalence of AGD in AD cases with severe neurofibrillary pathology may reflect difficulties in distinguishing the lesions.

Several studies have shown the association of AGD with other neurodegenerative diseases, including AD, Pick's Disease, tangle-only dementia, Creutzeldt-Jacob disease, Parkinson's Disease, Dementia with Lewy body, amyotrophic lateral sclerosis, PSP, and CBD.^4,5,26,28,43,47,63^ AGD prevalence in these cases is apparently higher than expected for age-matched individuals. For instance, AGD is frequently observed in CBD (57.7% versus 30-40% age-matched subjects) and PSP cases.^6,7,28,46,64^ These findings suggest that AGD shares common genetic or pathogenic features with PSP and CBD. Along the same lines, Tatsumi et al. described a frequency of 100% AGs in CBD cases.^43^ Despite the high frequency of AG in PSP and CBD cases, a high prevalence of PSP and CBD pathology has not been observed in primary AGD cases, even in large cohorts.^4,57^

Recent studies have shown a high prevalence (40-60%) of hyperphosphorylated transactive response DNA-binding protein with 43kD (TPD-43) in AGD cases, mainly in the limbic system.^65,66^ The clinical significance of TDP-43 pathology in the brains of patients with various neurodegenerative disorders remains uncertain.^67-71^

**Genetics.** AGD is a predominantly sporadic disease. Recent studies described two MAPT mutations causing pathological features consistent with AGD.^20,72^ A study published in 2008 demonstrated MAPT S305I mutation in a case with neuropathological diagnosis of AGD, while the most recent report described S305S mutation in a Scandinavian family where two siblings had neuropathological features resembling AGD. These reports describe common clinical features of early memory problems and behavioral changes. However, the S305S mutation was also associated with frontotemporal dementia with parkinsonism and PSP in previous studies.^73^ Villela et al., in a study investigating DNA copy number variations (CNV) in 28 AGD cases, identified rare constitutive CNVs in these individuals. This finding suggested a CNV at 17p^13^.^2^ as a strong candidate for causing AGD.^74^ Studies have demonstrated the role of CNV in the etiology of several neuropsychiatric disorders.^75^ Unlike other 4R tauopathies such as PSP and CBD, there are controversies over the relationship of the tau H1 haplotype with positive and negative results.^18,50^

The apolipoprotein E (APOE) ε2 allele was associated with increased risk of AGD in one study, but further studies failed to confirm this relationship.^64,76^

**AGD spread in experimental models.** The progressive aggregation of a particular protein in a stereotypical pattern is a common characteristic of all neurodegenerative disorders. Recent studies showed that a group of proteins, including abnormal tau protein, can trigger self-propagation in a prion-like fashion. These proteins share with prions the molecular properties of nucleation, templating, growth, multiplication and spread.^77^

Clavaguera et al. showed that intracerebral injection of brain extracts containing aggregated tau, including AGD, induces tauopathy in tau-transgenic host mice, and the induced tau lesions propagate systematically from the injection site to axonally connected areas suggesting neuronal uptake, transport and release of tau seeds. However, these mice did not show signs of neurodegeneration, suggesting that the molecular species responsible for propagation and neurotoxicity could be different.^78^

Ahmed et al. hypothesized that the spread of tau protein aggregates depends on synaptic connectivity rather than spatial proximity.^79^ Additionally, *in vitro* studies showed that the intracellular entry of tau filaments occurs through endocytosis.^80,81^

In a recent study, Dujardin et al. demonstrated a differential tau spread between wild-type and mutant mice suggesting distinct mechanism for genetic and sporadic forms of tauopathies.^82^ Sanders et. al also demonstrated spread of abnormal tau in cell cultures exposed to homogenates of human brains with AGD.^83^

## Conclusion.

Clinicopathological studies have recognized AGD as a distinctive and highly frequent neurodegenerative disease. Aging is a well-established risk factor for AGD. To date, AGD diagnosis has been exclusively *post-mortem*. The most typical clinical phenotype associated to AGD is slowly progressive mild cognitive impairment with a high frequency of neuropsychiatric symptoms that sometimes might precede the cognitive component. Early and prominent involvement of the limbic system could explain the presence of neuropsychiatric symptoms.

The reasons why AGD does not develop with prominent clinical features have yet to be clarified. Recent evidence of lack of tau acetylation in AGD suggest a protective role of this entity against spread of other neurodegenerative conditions, particularly Alzheimer's disease and is a hypothesis currently being investigated.

## References

[1] Grinberg LT, Heinsen H (2009). Argyrophilic grain disease: An update about a frequent cause of
dementia. Dement Neuropsychol.

[2] Thal DR, Schultz C, Botez G (2005). The impact of argyrophilic grain disease on the development of
dementia and its relationship to concurrent Alzheimer's disease-related
pathology. Neuropathol Appl Neurobiol.

[3] Pham CT, de Silva R, Haik S (2011). Tau-positive grains are constant in centenarians'
hippocampus. Neurobiol Aging.

[4] Braak H, Braak E (1998). Argyrophilic grain disease: frequency of occurrence in different
age categories and neuropathological diagnostic criteria. J Neural transm.

[5] Jellinger KA (1998). Dementia with grains (argyrophilic grain disease). Brain pathol.

[6] Saito Y, Ruberu NN, Sawabe M (2004). Staging of argyrophilic grains: an age-associated
tauopathy. J Neuropathol Exp Neurol.

[7] Ding ZT, Wang Y, Jiang YP (2006). Argyrophilic grain disease: frequency and neuropathology in
centenarians. Acta Neuropathologica.

[8] Tolnay M, Clavaguera F (2004). Argyrophilic grain disease: a late-onset dementia with
distinctive features among tauopathies. Neuropathology.

[9] Tolnay M, Schwietert M, Monsch AU, Staehelin HB, Langui D, Probst A (1997). Argyrophilic grain disease: distribution of grains in patients
with and without dementia. Acta Neuropathol.

[10] Ishihara K, Araki S, Ihori N (2005). Argyrophilic grain disease presenting with frontotemporal
dementia: a neuropsychological and pathological study of an autopsied case
with presenile onset. Neuropathology.

[11] Grinberg LT, Ferretti RE, Farfel JM (2007). Brain bank of the Brazilian aging brain study group - a milestone
reached and more than 1,600 collected brains. Cell Tissue Bank.

[12] Braak H, Braak E (1987). Argyrophilic grains: characteristic pathology of cerebral cortex
in cases of adult onset dementia without Alzheimer changes. Neurosci Lett.

[13] Josephs KA, Whitwell JL, Parisi JE (2008). Argyrophilic grains: a distinct disease or an additive
pathology?. Neurobiol Aging.

[14] Jicha GA, Petersen RC, Knopman DS (2006). Argyrophilic grain disease in demented subjects presenting
initially with amnestic mild cognitive impairment. J Neuropathol Exp Neurol.

[15] Kovacs GG, Milenkovic I, Wohrer A (2013). Non-Alzheimer neurodegenerative pathologies and their
combinations are more frequent than commonly believed in the elderly brain:
a community-based autopsy series. Acta Neuropathol.

[16] Ilieva EV, Kichev A, Naudi A, Ferrer I, Pamplona R, Portero-Otin M (2011). Mitochondrial dysfunction and oxidative and endoplasmic reticulum
stress in argyrophilic grain disease. J Neuropathol Exp Neurol.

[17] Ferrer I, Barrachina M, Tolnay M (2003). Phosphorylated protein kinases associated with neuronal and glial
tau deposits in argyrophilic grain disease. Brain Pathol.

[18] Miserez AR, Clavaguera F, Monsch AU, Probst A, Tolnay M (2003). Argyrophilic grain disease: molecular genetic difference to other
four-repeat tauopathies. Acta Neuropathol.

[19] Botez G, Probst A, Ipsen S, Tolnay M (1999). Astrocytes expressing hyperphosphorylated tau protein without
glial fibrillary tangles in argyrophilic grain disease. Acta Neuropathol.

[20] Kovacs GG, Pittman A, Revesz T (2008). MAPT S305I mutation: implications for argyrophilic grain
disease. Acta Neuropathol.

[21] Tolnay M, Sergeant N, Ghestem A (2002). Argyrophilic grain disease and Alzheimer's disease are
distinguished by their different distribution of tau protein
isoforms. Acta Neuropathol.

[22] Grinberg LT, Wang X, Wang C (2013). Argyrophilic grain disease differs from other tauopathies by
lacking tau acetylation. Acta Neuropathol.

[23] Thal DR, Capetillo-Zarate E, Galuske RA (2008). Tracing of temporo-entorhinal connections in the human brain:
cognitively impaired argyrophilic grain disease cases show dendritic
alterations but no axonal disconnection of temporo-entorhinal association
neurons. Acta Neuropathol.

[24] Braak H, Braak E (1991). Neuropathological stageing of Alzheimer-related
changes. Acta Neuropathol.

[25] Braak H, Braak E (1989). Cortical and subcortical argyrophilic grains characterize a
disease associated with adult onset dementia. Neuropathol Appl Neurobiol.

[26] Ikeda K, Akiyama H, Kondo H, Haga C (1995). A study of dementia with argyrophilic grains. Possible
cytoskeletal abnormality in dendrospinal portion of neurons and
oligodendroglia. Acta Neuropathol.

[27] Itagaki S, McGeer PL, Akiyama H (1989). A case of adult-onset dementia with argyrophilic
grains. Ann Neurol.

[28] Martinez-Lage P, Munoz DG (1997). Prevalence and disease associations of argyrophilic grains of
Braak. J Neuropathol Exp Neurol.

[29] Masliah E, Hansen LA, Quijada S (1991). Late onset dementia with argyrophilic grains and subcortical
tangles or atypical progressive supranuclear palsy?. Ann Neurol.

[30] Tolnay M, Mistl C, Ipsen S, Probst A (1998). Argyrophilic grains of Braak: occurrence in dendrites of neurons
containing hyperphosphorylated tau protein. Neuropathol Appl Neurobiol.

[31] Tolnay M, Calhoun M, Pham HC, Egensperger R, Probst A (1999). Low amyloid (Abeta) plaque load and relative predominance of
diffuse plaques distinguish argyrophilic grain disease from Alzheimer's
disease. Neuropathol Appl Neurobiol.

[32] Yamada T, McGeer PL, McGeer EG (1992). Some immunohistochemical features of argyrophilic grain dementia
with normal cortical choline acetyltransferase levels but extensive
subcortical pathology and markedly reduced dopamine. J Geriatr Psychiatry Neurol.

[33] Tolnay M, Spillantini MG, Goedert M, Ulrich J, Langui D, Probst A (1997). Argyrophilic grain disease: widespread hyperphosphorylation of
tau protein in limbic neurons. Acta Neuropathol.

[34] Tolnay M, Probst A (1998). Ballooned neurons expressing alphaB-crystallin as a constant
feature of the amygdala in argyrophilic grain disease. Neurosci Lett.

[35] Adachi T, Saito Y, Hatsuta H (2010). Neuropathological asymmetry in argyrophilic grain
disease. J Neuropathol Exp Neurol.

[36] Saito Y, Murayama S (2007). Neuropathology of mild cognitive impairment. Neuropathology.

[37] Asaoka T, Tsuchiya K, Fujishiro H (2010). Argyrophilic grain disease with delusions and hallucinations: a
pathological study. Psychogeriatrics.

[38] Togo T, Isojima D, Akatsu H (2005). Clinical features of argyrophilic grain disease: a retrospective
survey of cases with neuropsychiatric symptoms. Am J Geriatr Psychiatry.

[39] Ikeda K, Akiyama H, Arai T, Matsushita M, Tsuchiya K, Miyazaki H (2000). Clinical aspects of argyrophilic grain disease. Clin Neuropathol.

[40] Tsuchiya K, Mitani K, Arai T (2001). Argyrophilic grain disease mimicking temporal Pick's disease: a
clinical, radiological, and pathological study of an autopsy case with a
clinical course of 15 years. Acta Neuropathol.

[41] Saito Y, Nakahara K, Yamanouchi H, Murayama S (2002). Severe involvement of ambient gyrus in dementia with
grains. J Neuropathol Exp Neurol.

[42] Grau-Rivera O, Gelpi E, Rey MJ (2013). Prominent psychiatric symptoms in patients with Parkinson's
disease and concomitant argyrophilic grain disease. J Neurol.

[43] Tatsumi S, Mimuro M, Iwasaki Y (2014). Argyrophilic grains are reliable disease-specific features of
corticobasal degeneration. J Neuropathol Exp Neurol.

[44] Maurage CA, Sergeant N, Schraen-Maschke S (2003). Diffuse form of argyrophilic grain disease: a new variant of
four-repeat tauopathy different from limbic argyrophilic grain
disease. Acta Neuropathol.

[45] Rascovsky K, Hodges JR, Knopman D (2011). Sensitivity of revised diagnostic criteria for the behavioural
variant of frontotemporal dementia. Brain.

[46] Knopman DS, Parisi JE, Salviati A (2003). Neuropathology of cognitively normal elderly. J Neuropathol Exp Neurol.

[47] Ferrer I, Santpere G, van Leeuwen FW (2008). Argyrophilic grain disease. Brain.

[48] Goedert M (2011). Introduction to the tauopathies in Neurodegeneration: the molecular
pathology of dementia and movement disorders.

[49] Spillantini MG, Goedert M (2013). Tau pathology and neurodegeneration. Lancet Neurol.

[50] Togo T, Sahara N, Yen SH (2002). Argyrophilic grain disease is a sporadic 4-repeat
tauopathy. J Neuropathol Exp Neurol.

[51] Higuchi M T, Lee VMY (2002). Tau Protein And Tauopathy in Neuropsychopharmacology.

[52] Clavaguera F, Grueninger F, Tolnay M (2014). Intercellular transfer of tau aggregates and spreading of tau
pathology: Implications for therapeutic strategies. Neuropharmacology.

[53] Cohen TJ, Guo JL, Hurtado DE (2011). The acetylation of tau inhibits its function and promotes
pathological tau aggregation. Nat Commun.

[54] Togo T, Dickson DW (2002). Ballooned neurons in progressive supranuclear palsy are usually
due to concurrent argyrophilic grain disease. Acta Neuropathol.

[55] Seubert P, Mawal-Dewan M, Barbour R (1995). Detection of phosphorylated Ser262 in fetal tau, adult tau, and
paired helical filament tau. J Biol Chem.

[56] Ramirez RR CI, Fumadó J (2011). Demencias Degenerativas infrequentes in Enfermedad de Alzheimer y otras
demencias.

[57] Probst A, Tolnay M (2002). Argyrophilic grain disease (AgD), a frequent and largely
underestimated cause of dementia in old patients. Rev Neurol (Paris).

[58] Hof PR, Bouras C, Constantinidis J, Morrison JH (1990). Selective disconnection of specific visual association pathways
in cases of Alzheimer's disease presenting with Balint's
syndrome. J Neuropathol Exp Neurol.

[59] Braak H, Braak E (1991). Neuropathological stageing of Alzheimer-related
changes. Acta Neuropathol.

[60] Davis D, Ross GW, Petrovitch H (1997). Quantification of argyrophilic grains in hippocampal CA-1 of aged
Japonese- American men. J Neuropathol Exp Neurol.

[61] Schultz C, Koppers D, Sassin I, Braak E, Braak H (1998). Cytoskeletal alterations in the human tuberal hypothalamus
related to argyrophilic grain disease. Acta Neuropathol.

[62] Ishizawa T, Ko LW, Cookson N, Davias P, Espinoza M, Dickson DW (2002). Selective neurofibrillary degeneration of the hippocampal CA2
sector is associated with four-repeat tauopathies. J Neuropathol Exp Neurol.

[63] Yokota O, Tsuchiya K, Noguchi Y (2007). Coexistence of amyotrophic lateral sclerosis and argyrophilic
grain disease: a non-demented autopsy case showing circumscribed temporal
atrophy and involvement of the amygdala. Neuropathology.

[64] Togo T, Cookson N, Dickson DW (2002). Argyrophilic grain disease: neuropathology, frequency in a
dementia brain bank and lack of relationship with apolipoprotein
E. Brain Pathol.

[65] Fujishiro H, Uchikado H, Arai T (2009). Accumulation of phosphorylated TDP-43 in brains of patients with
argyrophilic grain disease. Acta Neuropathol.

[66] Arnold SJ, Dugger BN, Beach TG (2013). TDP-43 deposition in prospectively followed, cognitively normal
elderly individuals: correlation with argyrophilic grains but not other
concomitant pathologies. Acta Neuropathol.

[67] Amador-Ortiz C, Lin WL, Ahmed Z (2007). TDP-43 immunoreactivity in hippocampal sclerosis and Alzheimer's
disease. Ann Neurol.

[68] Arai T, Mackenzie IR, Hasegawa M (2009). Phosphorylated TDP-43 in Alzheimer's disease and dementia with
Lewy bodies. Acta Neuropathol.

[69] Nakashima-Yasuda H, Uryu K, Robinson J (2007). Co-morbidity of TDP-43 proteinopathy in Lewy body related
diseases. Acta neuropathologica.

[70] Uryu K, Nakashima-Yasuda H, Forman MS (2008). Concomitant TAR-DNA-binding protein 43 pathology is present in
Alzheimer disease and corticobasal degeneration but not in other
tauopathies. J Neuropathol Exp Neurol.

[71] Schwab C, Arai T, Hasegawa M, Yu S, McGeer PL (2008). Colocalization of transactivation-responsive DNA-binding protein
43 and huntingtin in inclusions of Huntington disease. J Neuropathol Exp Neurol.

[72] Ronnback A, Nennesmo I, Tuominen H, Grueninger F, Viitanen M, Graff C (2014). Neuropathological characterization of two siblings carrying the
MAPT S305S mutation demonstrates features resembling argyrophilic grain
disease. Acta Neuropathol.

[73] Skoglund L, Viitanen M, Kalimo H (2008). The tau S305S mutation causes frontotemporal dementia with
parkinsonism. Eur J Neurol.

[74] Villela D, Kimura L, Schlesinger D (2013). Germline DNA copy number variation in individuals with
Argyrophilic grain disease reveals CTNS as a plausible candidate
gene. Genet Mol Biol.

[75] Cook EH Jr., Scherer SW (2008). Copy-number variations associated with neuropsychiatric
conditions. Nature.

[76] Ghebremedhin E, Schultz C, Botez G (1998). Argyrophilic grain disease is associated with apolipoprotein E
epsilon 2 allele. Acta Neuropathol.

[77] Jucker M, Walker LC (2013). Self-propagation of pathogenic protein aggregates in
neurodegenerative diseases. Nature.

[78] Clavaguera F, Akatsu H, Fraser G (2013). Brain homogenates from human tauopathies induce tau inclusions in
mouse brain. Proc Natl Acad Sci U S A.

[79] Ahmed Z, Cooper J, Murray TK (2014). A novel in vivo model of tau propagation with rapid and
progressive neurofibrillary tangle pathology: the pattern of spread is
determined by connectivity, not proximity. Acta Neuropathol.

[80] Frost B, Jacks RL, Diamond MI (2009). Propagation of tau misfolding from the outside to the inside of a
cell. J Biol Chem.

[81] Guo JL, Lee VM (2011). Seeding of normal Tau by pathological Tau conformers drives
pathogenesis of Alzheimer-like tangles. J Biol Chem.

[82] Dujardin S, Lecolle K, Caillierez R (2014). Neuron-to-neuron wild-type Tau protein transfer through a
trans-synaptic mechanism: relevance to sporadic tauopathies. Acta Neuropathol Commun.

[83] Sanders DW, Kaufman SK, DeVos SL (2014). Distinct Tau Prion Strains Propagate in Cells and Mice and Define
Different Tauopathies. Neuron.

